# Recurrent pancreatitis secondary to hypertriglyceridemia: A case report and case review

**DOI:** 10.1002/ccr3.7811

**Published:** 2023-09-19

**Authors:** Mohamed Nazeef, Sagar Devkota, Sumnima Mainali, Yubaraj Thapa, Sristi Upadhyay, Priyanka Yadav, Jeena Shrestha

**Affiliations:** ^1^ Department of Internal Medicine Kulhudhuffushi Regional Hospital Kulhudhuffushi Maldives; ^2^ Department of Anesthesiology and Critical Care Kulhushuffushi Regional Hospital Kulhudhuffushi Maldives; ^3^ Department of Obstetrics and Gynecology Kulhudhuffushi Regional Hospital Kulhudhuffushi Maldives; ^4^ Department of Pediatrics and Adolescent Medicine Kulhudhuffushi Regional Hospital Kulhudhuffushi Maldives; ^5^ Department of Obstetrics and Gynecology Nepal Medical College and Teaching Hospital Kathmandu Nepal; ^6^ Extern, The Family Doc Clinic and Urgent Care Dearborn Heights Michigan USA

**Keywords:** acute pancreatitis, hypertriglyceridemia, pregnancy, recurrence

## Abstract

**Key Clinical Message:**

Managing acute pancreatitis secondary to hypertriglyceridemia in pregnancy is challenging. The use of intravenous insulin along with lipid lowering drugs can be an option in settings where plasmapharesis and gene therapy are unavailable.

**Abstract:**

Acute pancreatitis secondary to hypertriglyceridemia is rare but various studies have highlighted it as the third most common cause following gallstones and alcohol consumption. Managing acute pancreatitis is always challenging; even more challenging during pregnancy. We present a case of a 31‐year– old female with a history of recurrent pancreatitis secondary to hypertriglyceridemia with a current episode of acute pancreatitis at 21 weeks of gestation.

## 1 INTRODUCTION

Acute pancreatitis is a grave medical condition with significant morbidity and mortality, and its diagnosis and severity are based on revised the Atlanta classification (2012).^1^ Known to be associated with various risk factors and etiologies, gallstones and alcohol consumption are the most common ones accounting for 30%–40% of the cases.^2^ Hypertriglyceridemia, though rare, is known to be third most common cause of acute pancreatitis associated with almost 1%–35% of all the cases and up to 56% of acute pancreatitis during pregnancy.^3^, ^4^

The risk of acute pancreatitis progressively increases with increase in triglyceride levels with marked increment with levels more than 1000 mg/dL.^5^, ^6^, ^7^, ^8^, ^9^ The level greater than 1000 mg/dL is associated with 5% risk of developing acute pancreatitis, while the risk increases to 10%–20% as the level goes beyond 2000 mg/dL.^10^ The treatment options include supportive care including prompt fluid resuscitation, pain management, and plasma lipid lowering therapies such as statins, fibrates, insulin, heparin, apheresis, and hemofiltration.^11^

We present a case of a 31‐year– old female with a history of recurrent episodes of pancreatitis with persistently elevated triglyceride levels with acute pancreatitis secondary to marked hypertriglyceridemia at 21 weeks of gestation.

## 2 CASE REPORT

**FIGURE 1:**
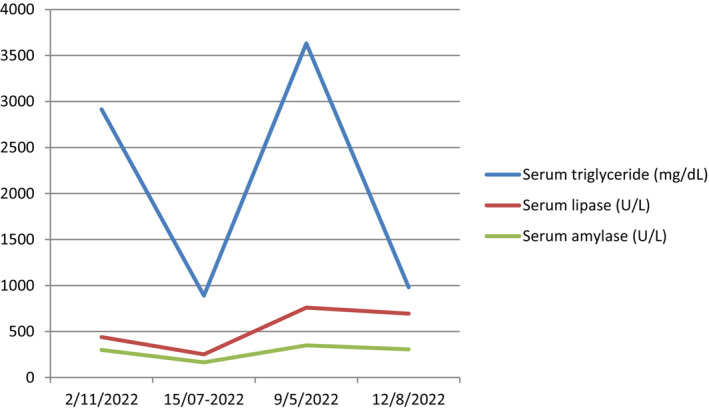
Laboratory findings of previous episodes.

A 31‐year–old female with a body mass index of 21 kg/m^2^ at 21 weeks of gestation presented to emergency department with complaints of a sudden onset of abdominal pain associated with multiple episodes of vomiting for 1 day. She denied any history of bleeding per vaginum and had normal bowel and bladder habits. She had a past history of recurrent pancreatitis (a total of 12 episodes since 2016) secondary to hypertriglyceridemia with the last episode being 6 months back (Figure 1). During her last episode, she was managed with intravenous insulin and discharged with insulin (glargine), omega‐2‐fatty acids, and rosuvastatin. Statins was stopped after she got pregnant. Her husband reported poor compliance to medications.

**FIGURE 2:**
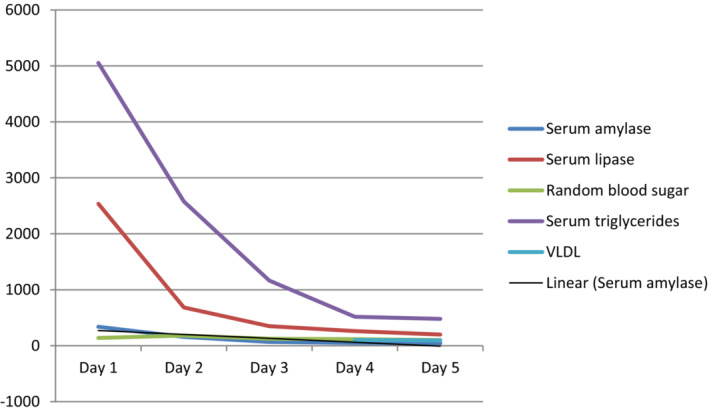
Laboratory findings during the episode.

The patient's family history was not significant and she denied taking alcohol or any other medications. She denied any history of decreased fetal movements. On examination, she was restless due to pain and was dehydrated, and her vitals were all within normal limits except for tachycardia and hypotension (heart rate in the range of 110–130 beats per minute and blood pressure in the range of 90–110/50–60 mm Hg). Her respiratory and cardiovascular examinations were normal. On palpation of abdomen, there was generalized tenderness along with guarding. Blood investigations revealed normal total blood counts, hypocalcaemia, and hypokalemia. Marked elevation of serum amylase (339 U/L) and serum lipase (2537 U/L) along with a deranged lipid profile with a total cholesterol level of 666 mg/dL and triglyceride level of 5054 mg/dL was noted. The level of glycated hemoglobin (HbA1C) was within normal limits. Urine analysis was also within normal limits. The thyroid function test was also normal. Arterial blood gas analysis revealed a pH of 7.379 with pO_2_ of 110 mm Hg and pCO_2_ of 30.8 mm Hg. Ultrasonography of abdomen was done, which revealed grossly enlarged pancreas with a dilated main pancreatic duct with no evidence of gallstones and mild peri‐pancreatic collection, suggestive of acute pancreatitis. Pelvic ultrasonography revealed single, live, intra‐uterine fetus of approximately 335 g at 20 weeks of gestation with a heart rate of 141 beats per minute. The patient was managed in the intensive care unit with a diagnosis of mild acute pancreatitis at 21 weeks of gestation secondary to hypertriglyceridemia with hypocalcaemia with hypokalemia with a BISAP^12^ score of 1 and a Ranson score^13^ 0 at the time of admission. Aggressive fluid resuscitation with 0.9% normal saline with potassium supplementation was done to target urine output of 0.5–1 mL/kg/h. Calcium gluconate was given for correction of hypocalcaemia and intravenous paracetamol was given three times a day along with intravenous tramadol as rescue for pain management. Intravenous insulin was started with monitoring of blood sugar accordingly. Omega‐3‐fatty acids were also given as anti‐oxidants. However, insulin alone did not reduce the lipid levels satisfactorily; oral lipid lowering agent (gemfibrozil) was started after discussion regarding its teratogenicity (pregnancy category C) as there was no response to other lipid lowering agents (During previous episodes, she was given fenofibrate). The serum lipase and serum triglycerides levels gradually reduced, and the patient got symptomatically better on the 4th day of ICU admission (Figure 2).

Multidisciplinary approach was done involving obstetrician and pediatrician. Fetal heart rate was monitored every 6 h and the biophysical profile was monitored every alternate day till admission. On the 5th day of admission, the patient was discharged on request with subcutaneous insulin glargine and oral medications (gemfibrozil and omega‐3‐fatty acids) and was advised for regular follow‐up. She came to the outpatient department for follow‐up at 2 weeks, and during follow up, all her laboratory reports were within normal limits. Her obstetric scan revealed single live intrauterine fetus at 22 weeks of gestation with normal cardiac activity.

## 3 DISCUSSION

Hypertriglyceridemia and acute pancreatitis are known to be associated, both as a causative factor and also as an epiphenomenon.^14^ Hypertriglyceridemia is known to be a possible etiology for acute pancreatitis due to genetic causes in 5% of the cases^15^ and more often secondary to other causes like diabetes, obesity, pregnancy, unhealthy dietary habit, hypothyroidism, alcohol, sepsis, renal failure, and medications like estrogen or steroids.^16^ In our case, the patient denied any significant family history and was not taking any other medications other than glargine and fibrates (which was stopped after she got pregnant).

Treatment options include dietary modification (reduced calories), weight reduction, and lipid lowering agents. Fibrates reduce plasma triglyceride levels up to 50% and increase high density lipoprotein (HDL) by 20%. Statins reduce cholesterol by inhibiting hydroxymethylglutaryl CoA reductase, thereby reducing coronary artery disease and end organ damage in diabetes. Similarly, omega‐3 fatty acids are also known to reduce plasma triglycerides by 20% when used in combination with other lipid lowering agents.^17^, ^18^ Various anti‐oxidants like selenium, beta‐carotene, vitamin C, and vitamin E are known to reduce recurrent episodes of pancreatitis by protecting the free‐radical induced pancreatic acinar damage.^19^ Recent advances in managing hypertriglyceridemia include plasmapharesis, insulin, and heparin in combination and lipoprotein lipase gene therapy.^20^

In our case, the patient was advised for dietary modifications and given omega‐3‐fatty acids and fibrates at each of her previous episodes but due to poor compliance to medications, she had recurrent bouts of acute pancreatitis. Insulin glargine was added on her last ICU admission as there was reduced response to other medications. During this episode, insulin along with gemfibrozil^21^ was started after careful discussion regarding its teratogenicity. Plasmapharesis and gene therapy were not available at our center, and the patient refused for referral to higher centers. She was discharged on long acting insulin and oral lipid lowering agents and advised for regular follow‐up.

## 4 CONCLUSION

Management of acute pancreatitis in pregnancy is always challenging and requires a multi‐disciplinary approach to reduce the morbidity and mortality associated with it. Insulin with heparin, plasmapheresis, and gene therapy are some of the very novel modalities of treatment during pregnancy but in limited resource settings, insulin along with lipid lowering agents like fibrates (considered safe after first trimester) can be an option after thorough discussion with the patient regarding its teratogenicity.

## AUTHOR CONTRIBUTIONS

**Mohamed Nazeef:** Conceptualization; methodology; writing – review and editing. **Sagar Devkota:** Conceptualization; formal analysis; investigation; methodology; resources; writing – original draft; writing – review and editing. **Sumnima Mainali:** Investigation; methodology; supervision; writing – review and editing. **Yubaraj Thapa:** Resources; writing – review and editing. **Sristi Upadhyay:** Writing – review andediting. **Priyanka Yadav:** Writing – review and editing. **Jeena Shrestha:** Writing – review and editing.

## FUNDING INFORMATION

No financial burden was on the patient.

## CONFLICT OF INTEREST STATEMENT

None.

### ETHICS STATEMENT

Ethical approval is not required for a case report at our hospital.

### CONSENT

Written informed consent was obtained from the patient to publish this report in accordance with the journal's patient consent policy.

## Data Availability

None.
